# Constitutive CCND1/CDK2 Activity Substitutes for p53 Loss, or MYC or Oncogenic RAS Expression in the Transformation of Human Mammary Epithelial Cells

**DOI:** 10.1371/journal.pone.0053776

**Published:** 2013-02-04

**Authors:** Damian J. Junk, Rocky Cipriano, Martha Stampfer, Mark W. Jackson

**Affiliations:** 1 Department of Pathology, Case Western Reserve University, Cleveland, Ohio, United States of America; 2 Life Sciences Division, Lawrence Berkeley National Laboratory, Berkeley, California, United States of America; 3 Case Comprehensive Cancer Center, Case Western Reserve University, Cleveland, Ohio, United States of America; Wayne State University School of Medicine, United States of America

## Abstract

Cancer develops following the accumulation of genetic and epigenetic alterations that inactivate tumor suppressor genes and activate proto-oncogenes. Dysregulated cyclin-dependent kinase (CDK) activity has oncogenic potential in breast cancer due to its ability to inactivate key tumor suppressor networks and drive aberrant proliferation. Accumulation or over-expression of cyclin D1 (CCND1) occurs in a majority of breast cancers and over-expression of CCND1 leads to accumulation of activated CCND1/CDK2 complexes in breast cancer cells. We describe here the role of constitutively active CCND1/CDK2 complexes in human mammary epithelial cell (HMEC) transformation. A genetically-defined, stepwise HMEC transformation model was generated by inhibiting p16 and p53 with shRNA, and expressing exogenous MYC and mutant RAS. By replacing components of this model, we demonstrate that constitutive CCND1/CDK2 activity effectively confers anchorage independent growth by inhibiting p53 or replacing MYC or oncogenic RAS expression. These findings are consistent with several clinical observations of luminal breast cancer sub-types that show elevated CCND1 typically occurs in specimens that retain wild-type p53, do not amplify MYC, and contain no RAS mutations. Taken together, these data suggest that targeted inhibition of constitutive CCND1/CDK2 activity may enhance the effectiveness of current treatments for luminal breast cancer.

## Introduction

Cancer cells arise through a stepwise process of transformation in which a normal cell acquires aberrant “hallmark” properties that include sustained proliferative signaling, inhibition of growth suppressors, replicative immortality, and resistance to cell death [1]. Studies over 25 years ago confirmed that normal murine cells could be transformed using a limited set of genetic manipulations including either c-MYC, polyoma large-T antigen, mutant p53 or adenoviral E1A combined with a hyperactive RAS gene [2]–[4]. Additional studies have demonstrated that more stringent tumor suppressive mechanisms govern human cell transformation, and human fibroblasts and epithelial cells differ in their requirements for transformation [5].

Effort over the past 30 years has produced a cell culture model in which normal, finite-lifespan human mammary epithelial cells (HMEC) can be cultured from reduction mammoplasty tissue [6]–[12]. Normal HMEC grown in culture first encounter a stress-induced senescence barrier called stasis, which is enforced by accumulation of p16, a cyclin-dependent kinase inhibitor that activates the RB family of tumor suppressors [8], [12]. However, when grown in the serum-free MCDB170 medium (commercial MEGM), rare post-selection cells emerge that no longer express p16 protein due to promoter methylation [7], [8]. Post-selection HMEC will continue to divide, incurring telomere erosion with each division, resulting in critically short telomeres that induce a second growth barrier due to telomere dysfunction. This barrier has been termed agonescence when p53 is functional and crisis in the absence of functional p53 [10]. Improved culture methods can now delay the onset of stasis in HMEC, permitting analysis of pre-stasis HMEC retaining functional p16 [12]. Thus, the role of p16-RB signaling can now be examined during HMEC transformation using pre-stasis cells. In addition, there exists a p16- and p53-independent senescence barrier engaged by dysregulated growth signals, termed oncogene induced senescence (OIS) [13], [14]. We have recently demonstrated that RAS-mediated OIS in HMEC requires TGF-β signaling, and can be prevented by suppressing TGF-β receptor activation or expressing MYC from a constitutive promoter [14]. Abrogation of TGF-β signaling not only allows HMEC to tolerate oncogenic RAS, but also confers the capacity for anchorage-independent growth (AIG), a property associated with malignancy [14].

Cyclins and cyclin-dependent kinases (CDK) are frequently dysregulated in cancer, and over-expression of cyclin D1 (CCND1) occurs in approximately 50% of breast cancers [15]–[18]. Over-expressed CCND1 binds to and activates CDK4 causing hyperphosphorylation of RB, which promotes cell cycle progression [19], [20]. In addition to CCND1/CDK4 complexes, over-expression of CCND1 also leads to accumulation of activated CCND1/CDK2 complexes in breast cancer cells [21]. Expression of a constitutively active CCND1/CDK2 fusion protein results in RB hyperphosphorylation on sites preferred by CDK4 and CDK2, confers resistance to TGF-β induced growth arrest in MMTVD1-K2 mouse tumor cells, causes sequestration and inhibition of p21, and induces AIG in mink lung epithelial cells [22], [23]. We have previously demonstrated that constitutive CCND1/CDK2 activity caused AIG in hTERT-immortalized post-selection HME-1 HMEC; however this activity alone could not transform non-immortalized post-selection HMEC to AIG suggesting that constitutive CCND1/CDK2 activity cooperated with other undefined events that had occurred only in the immortalized post-selection HME-1 [24].

Here we demonstrate that transformation to AIG of pre-stasis HMEC with functional p16 requires inhibition of the p16-RB axis, and that targeted suppression of p16 and p53 signaling allows for the cooperative transformation of HMEC by MYC and RAS to AIG. Importantly, we demonstrate that constitutive CCND1/CDK2 activity effectively replaces numerous individual components of transformation including p53 inhibition, MYC over-expression, or oncogenic RAS expression. Understanding the requirements of human breast epithelial cell transformation and the unique role of constitutive CCND1/CDK2 activity can guide the development of targeted strategies to treat breast cancer.

## Results

### A genetically-defined, stepwise transformation model of AIG in pre-stasis HMEC

**Figure 1:**
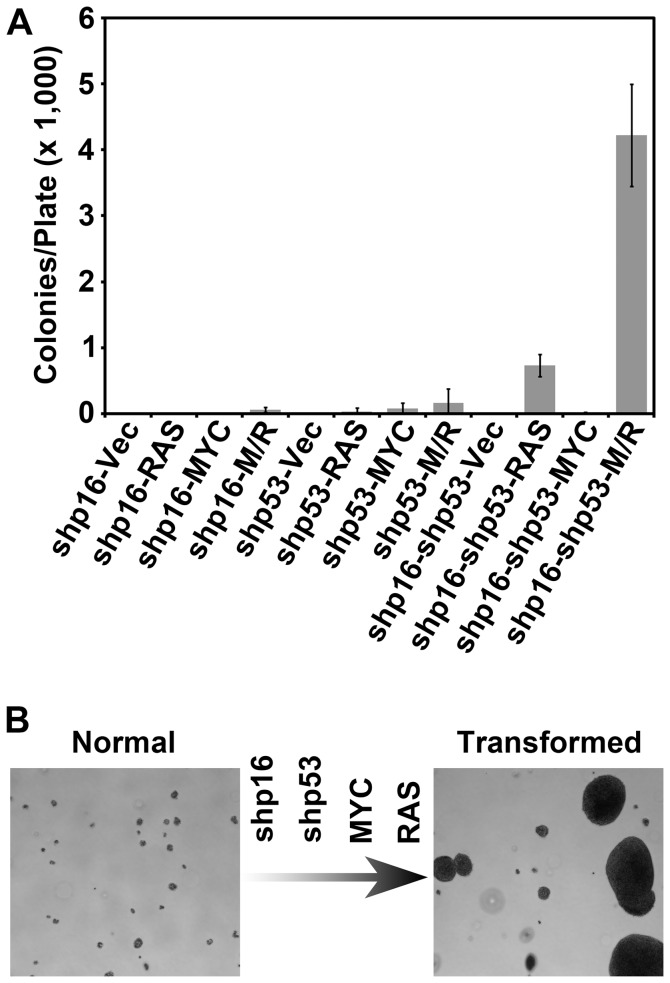
Generation of a genetically-defined, stepwise model of HMEC transformation. 48R expressing shRNA targeting p16 (shp16), p53 (shp53), or both (shp16-shp53) were further infected with control virus (Vec), or viruses encoding RAS alone (RAS), MYC alone (MYC), or MYC and RAS together (M/R) and assessed at passage 19 for AIG. (A) The bar graph represents the average colony number per plate of quadruplicates. Error bars represent the standard deviation. (B) Pictorial representation of the HMEC transformation model and growth in soft agar. Normal cells (left) are transformed (right) by sequential inactivation of p16 and p53 using shRNA, and over-expression of MYC and oncogenic RAS.

Improved culture methods permit the analysis of pre-stasis HMEC expressing functional p16. We previously developed a genetically-defined model of pre-stasis HMEC transformation to AIG utilizing viral infections to deliver shRNA to inactivate p16 and p53, and to express exogenous MYC, and oncogenic RAS [14]. Here we sought to determine if inhibition of the p16-RB axis is necessary for transformation to AIG in pre-stasis HMEC. Pre-stasis HMEC specimen 48R batch T (48R) cells were infected with viruses expressing shRNA targeting p16 (48R-shp16), p53 (48R-shp53), or p16 then p53 (48R-shp16-shp53), and selected with the appropriate antibiotics to remove uninfected cells. 48R-shp16, 48R-shp53, and 48R-shp16-shp53 populations were subsequently infected with retroviruses encoding RAS alone, MYC alone, MYC and RAS together, or a control retrovirus. Each of the twelve recombinant derivatives was plated in soft agar to assess AIG, a property associated with malignant transformation. Transformation of 48R cells to AIG was observed with inhibition of the tumor suppressors p16 and p53 and increased MYC and RAS oncogene expression, but any combination of these changes that did not include all four genetic events was insufficient to cause AIG (**Fig.** **1A**). These results demonstrate that disruption of the p16-RB signaling axis is required for transformation to AIG of 48R cells, and confirm the generation of a genetically-defined, stepwise model of HMEC transformation to AIG (**Fig.** **1B**). This finding is significant since most finite lifespan HMEC in current use, including commercially available cells, have inactive p16 due to promoter methylation [7], [8]. Therefore, the pre-stasis HMEC model is uniquely capable of determining how the p16-RB signaling axis regulates the transformation of HMEC.

### Constitutive CCND1/CDK2 activity replaces p53 inhibition in HMEC transformation to AIG

**Figure 2:**
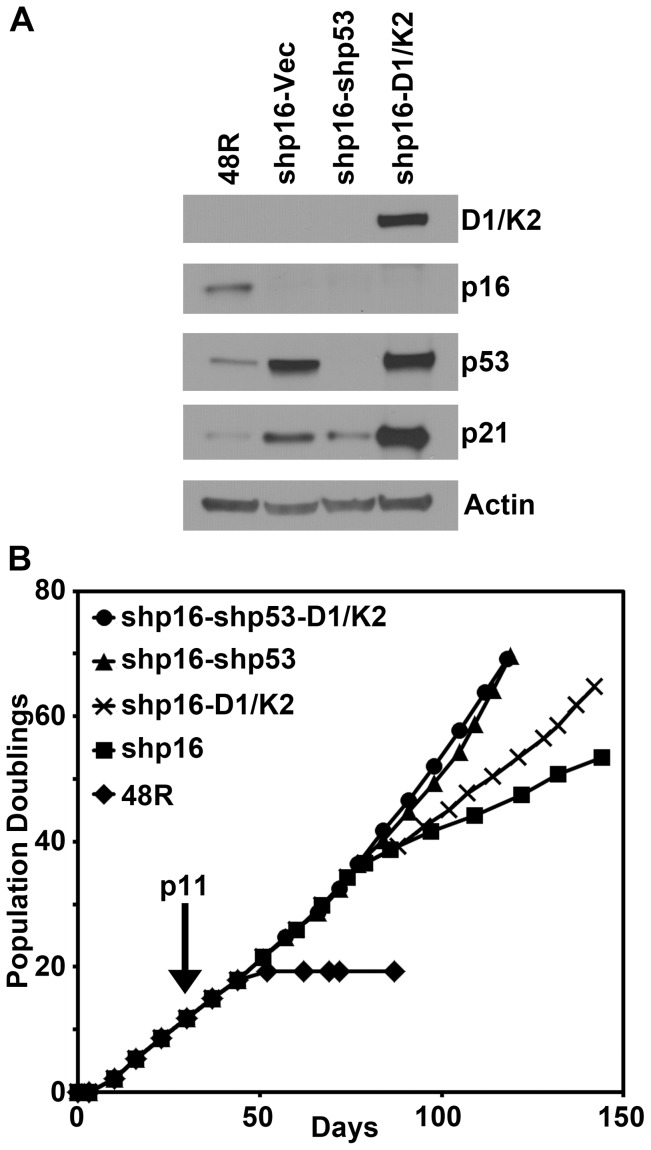
Constitutive CCND1/CDK2 activity enhances the growth of 48R-shp16 cells despite elevated p53 and p21. 48R-shp16 cells were infected with viruses encoding CCND1/CDK2 (D1/K2), a shRNA targeting p53 (shp53), or control virus (Vec). (A) Western blot analysis comparing parental 48R passage 11 to derivative cells. (B) Population doublings of the parental 48R (diamonds), 48R-shp16 (squares), 48R-shp16-D1/K2 (crosses), 48R-shp16-shp53 (triangles), and 48R-shp16-shp53-D1/K2 (circles) cells. Cells were grown from passage 6 at the origin and infected with shp16 at passage 11 indicated by the arrow.

A previous report demonstrated that CCND1/CDK2 complexes could bind, sequester, and inhibit p21 suggesting this complex may inhibit the p53-p21 axis [22]. Therefore, we sought to determine if constitutive CCND1/CDK2 activity could replace shp53 in our transformation model once p16 was suppressed. For this, 48R-shp16 cells were generated that expressed CCND1/CDK2 (48R-shp16-D1/K2) or infected with a control virus (48R-shp16-Vec); 48R-shp16-shp53 cells were used as a positive control. Western blot analysis confirmed expression of the CCND1/CDK2 fusion protein and appropriate inhibition of p16 and p53 in the shRNA-expressing cells compared to the parental 48R cells (**Fig.** **2A**) and the long-term growth of each 48R population was assessed. Parental 48R cells grew for 20 PD over 50 days before entering stasis (**Fig.** **2B**). As expected, ablation of p16 extended the growth of 48R-shp16 cells to 45 PD over 145 days (**Fig.** **2B**). Although the 48R-shp16-D1/K2 cells contained elevated levels of p53 and p21 (**Fig.** **2A**), these cells grew over 60 PD in 145 days (**Fig.** **2B**). The positive control 48R-shp16-shp53 cells grew to 70 PD in 120 days (**Fig.** **2B**). Additional expression of constitutive CCND1/CDK2 activity did not significantly alter the growth of the 48R-shp16-shp53 positive control cells (**Fig.** **2B**). These data demonstrate that constitutive CCND1/CDK2 activity allows for the growth of 48R-shp16 cells under conditions of elevated p53 and p21.

**Figure 3:**
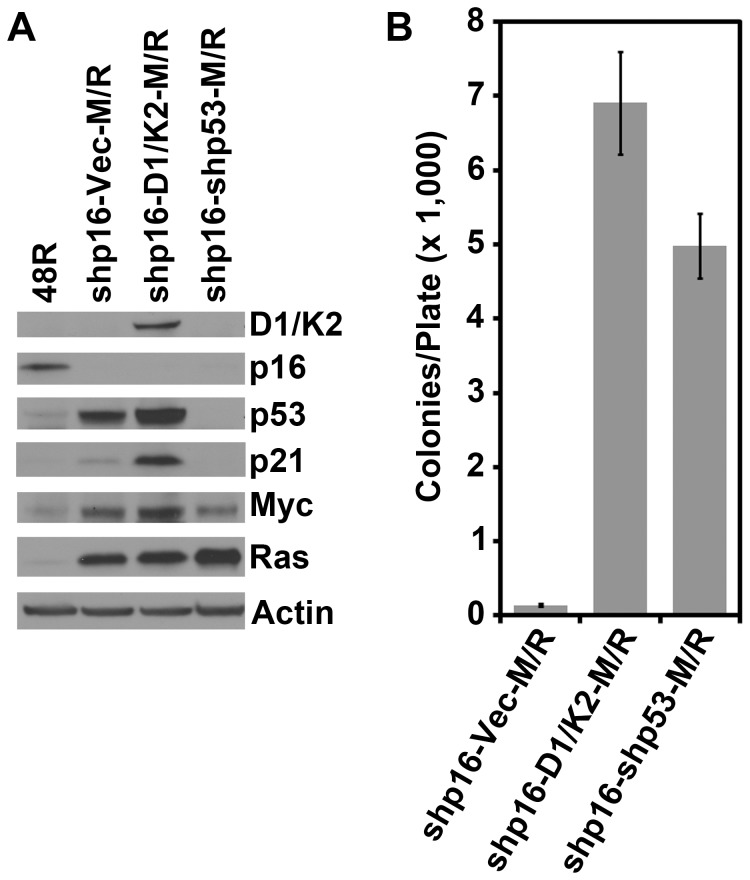
Constitutive CCND1/CDK2 activity replaces p53 inactivation in the transformation of HMEC. 48R-shp16 cells were infected with control virus (Vec), or viruses encoding CCND1/CDK2 (D1/K2), or shRNA targeting p53 (shp53), followed by virus encoding MYC and RAS (M/R). (A) Western blot analysis comparing parental 48R passage 11 to derivative cells. (B) Derivative cells from A were assessed for AIG. The bar graph represents the average colony number per plate of quadruplicates. Error bars represent the standard deviation.

Since the 48R-shp16-D1/K2 cells grew well despite elevated p53 and p21 protein levels, we sought to determine if constitutive CCND1/CDK2 activity could replace p53 inhibition in conferring AIG. 48R-shp16 cells were infected with viruses encoding CCND1/CDK2, shp53, or a control virus. These three derivatives were subsequently infected with retroviruses encoding MYC and RAS together generating 48R-shp16-Vec-M/R, 48R-shp16-D1/K2-M/R and 48R-shp16-shp53-M/R derivatives. Western blot analysis confirmed the knock-down of p16 and p53, and expression of CCND1/CDK2, MYC and RAS in the appropriate cell lines compared to the parental 48R cells (**Fig.** **3A**). Again, CCND1/CDK2 expression allowed elevation of p53 and p21 levels (**Fig.** **3A**). Each of the three derivatives was plated into soft agar to assess AIG. The 48R-shp16-D1/K2-M/R cells grew well in soft agar similarly to the positive control 48R-shp16-shp53-M/R cells (**Fig.** **3B**). These data suggest that although p53 and p21 are elevated in CCND1/CDK2 expressing 48R cells, constitutive CCND1/CDK2 activity inhibits the downstream effects of the p53-p21 axis, effectively replacing p53 inhibition in this transformation protocol.

### Constitutive CCND1/CDK2 activity can replace either MYC or RAS in HMEC transformation to AIG

**Figure 4:**
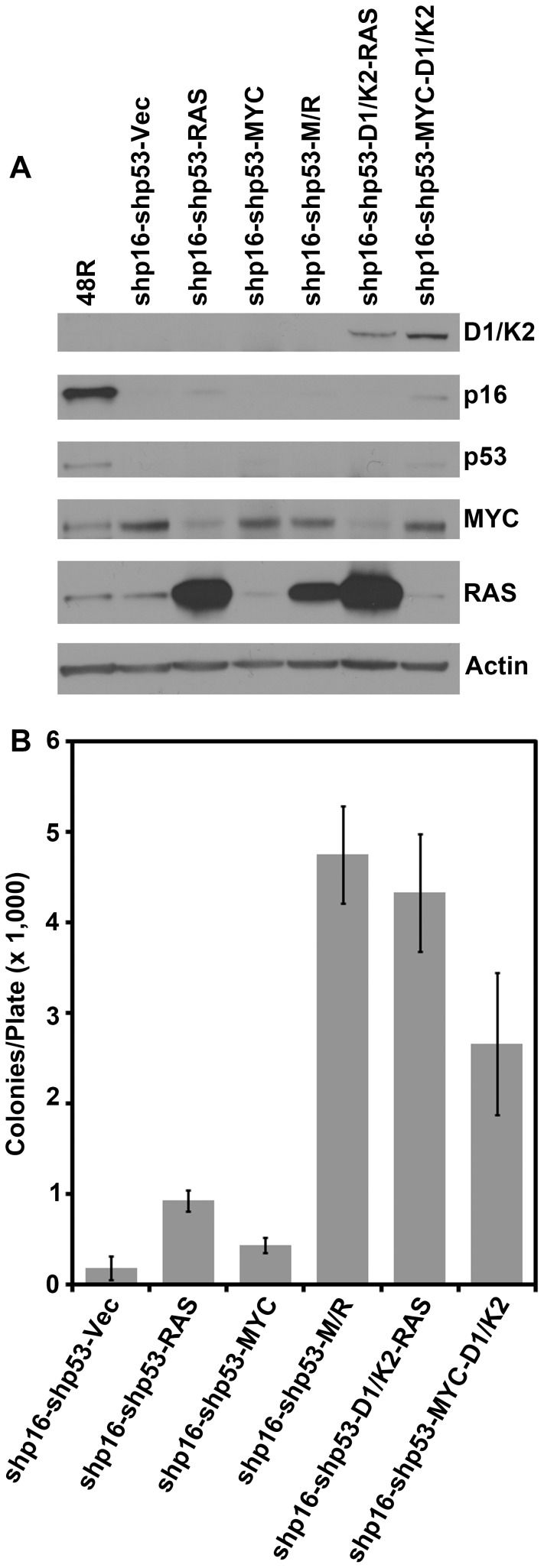
Constitutive CCND1/CDK2 activity replaces either MYC or RAS in the transformation of HMEC. 48R-shp16-shp53 cells were infected with control virus (Vec), virus encoding RAS alone (RAS), MYC alone (MYC), or MYC and RAS together (M/R). Additionally, 48R-shp16-shp53 cells were infected with viruses encoding CCND1/CDK2 (D1/K2) followed by RAS, or viruses encoding MYC followed by CCND1/CDK2 (D1/K2). (A) Western blot analysis comparing parental 48R passage 11 to derivative cells. (B) Each derivative was plated in soft agar to assess AIG. The bar graph represents the average colony number per plate of quadruplicates. Error bars represent the standard deviation.

We have previously reported that RAS expression engages a p16- and p53-independent OIS through enhanced TGF-β signaling [14]. Constitutive expression of MYC counteracts the TGF-β induced growth arrest, thereby allowing dysregulated RAS to drive HMEC transformation. Constitutive CCND1/CDK2 activity conferred resistance to TGF-β induced growth arrest in MMTVD1-K2 driven mouse mammary tumor cells, and CCND1 is a downstream effector of RAS [22], [23]. Therefore, we sought to determine if constitutive CCND1/CDK2 activity could replace MYC or RAS to drive HMEC transformation to AIG. 48R-shp16-shp53 cells were infected with retroviruses encoding RAS alone, MYC alone, MYC and RAS together, or control retroviruses to generate controls for the transformation protocol. Additionally, 48R-shp16-shp53 cells were infected with retroviruses encoding CCND1/CDK2 followed by RAS (to determine whether CCND1/CDK2 could replace MYC; 48R-shp16-shp53-D1/K2-RAS), or MYC followed by CCND1/CDK2 (to determine whether CCND1/CDK2 could replace RAS; 48R-shp16-shp53-MYC-D1/K2). Western blot analysis verified the expression of CCND1/CDK2, RAS, or MYC compared to the parental 48R cells (**Fig.** **4A**) and AIG was assessed. 48R-shp16-shp53 cells expressing vector control, RAS alone, or MYC alone demonstrated very low colony growth per plate (**Fig.** **4B**). The 48R-shp16-shp53-D1/K2-RAS cells (MYC replacement) grew as well as the positive control 48R-shp16-shp53-M/R cells, while the 48R-shp16-shp53-MYC-D1/K2 cells (RAS replacement) grew nearly as efficiently (**Fig.** **4B**). These results demonstrate that constitutive CCND1/CDK2 activity can efficiently replace two different oncogenes, MYC or RAS, to transform 48R-shp16-shp53 HMEC to AIG. Constitutive CCND1/CDK2 activity may replace MYC during transformation due to its ability to counteract TGF-β induced growth arrest induced by constitutive RAS signaling. Moreover, constitutive CCND1/CDK2 activity can replace RAS in the transformation protocol, albeit less efficiently.

### Constitutive CCND1/CDK2 activity effectively replaces only the single events shp53, MYC, or RAS in HMEC transformation to AIG

**Figure 5:**
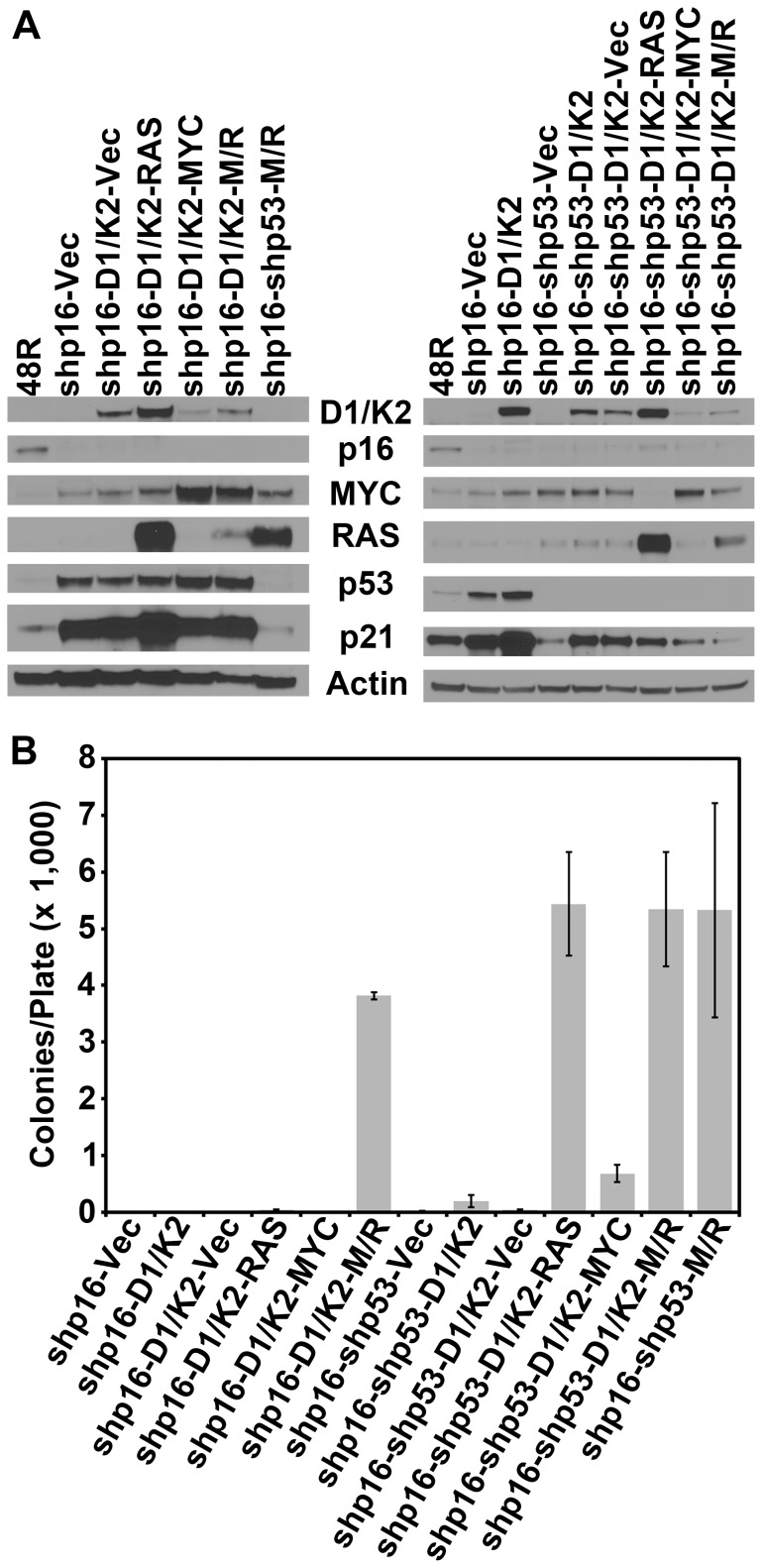
Constitutive CCND1/CDK2 activity does not replace multiple components of transformation. 48R-shp16-D1/K2 and 48R-shp16-shp53-D1/K2 cells were infected with control virus (Vec), virus encoding RAS alone (RAS), MYC alone (MYC), or MYC and RAS together (M/R). (A) Western blot analyses comparing parental 48R passage 11 to derivative cells. (B) Each derivative was plated in soft agar to assess AIG. The bar graph represents the average colony number per plate of quadruplicates. Error bars represent the standard deviation.

Our analyses demonstrate that constitutively active CCND1/CDK2 activity can substitute individually for shp53, MYC, or RAS expression; therefore we sought to determine if it could substitute for any combination of these events. 48R-shp16-D1/K2 and 48R-shp16-shp53-D1/K2 cells were infected with retroviruses expressing MYC alone, RAS alone, MYC and RAS together, or a control virus. Western blot analyses verified the expression of CCND1/CDK2, RAS, or MYC in the appropriate cell derivatives compared to parental 48R and control cells (**Fig.** **5A**). Constitutive CCND1/CDK2 activity caused accumulation of p53 except in cells expressing shp53, and accumulation of p21 regardless of p53 expression (**Fig.** **5A**). These data demonstrate that expression of CCND1/CDK2 induces p21 accumulation independent of the presence of p53 protein, likely due to its ability to bind and sequester p21 as previously reported [22]. The ability of constitutive CCND1/CDK2 activity to replace multiple components of the transformation protocol was tested by growth in soft agar. 48R-shp16-D1/K2 cells (shp53, MYC, and RAS replacement) showed no capacity for AIG equivalent to the 48R-shp16-Vec negative control cells (**Fig.** **5B**). 48R-shp16-D1/K2 cells expressing RAS alone (shp53 and MYC replacement) and MYC alone (shp53 and RAS replacement) did not grow anchorage independently equivalent to 48R-shp16-D1/K2-Vec control cells (**Fig.** **5B**). 48R-shp16-D1/K2 cells expressing MYC and RAS together (shp53 replacement) again demonstrated efficient agar growth (**Figs.** **3B** **and** **5B**). 48R-shp16-shp53-D1/K2 cells (MYC and RAS replacement) showed little growth in soft agar, while 48R-shp16-shp53-D1/K2-RAS cells (MYC replacement) grew well again (**Figs.** **4B** **and** **5B**). Interestingly, 48R-shp16-shp53-D1/K2-MYC cells (RAS replacement) showed a diminished capacity for AIG compared to the equivalent shp16-shp53-MYC-D1/K2 cells generated above (**Figs.** **4B** **and** **5B**). 48R-shp16-shp53-D1/K2-M/R cells showed an equivalent capacity for AIG to the 48R-shp16-shp53-M/R positive control cells (**Fig.** **5B**). These data suggest constitutive CCND1/CDK2 activity only replaces p53 inhibition or MYC or oncogenic RAS expression as a single component of the transformation protocol. In the case of RAS replacement the order of events affects the efficiency of AIG. Addition of constitutive CCND1/CDK2 activity does not enhance AIG of the 48R cells expressing shp16, shp53, MYC, and RAS.

**Figure 6:**
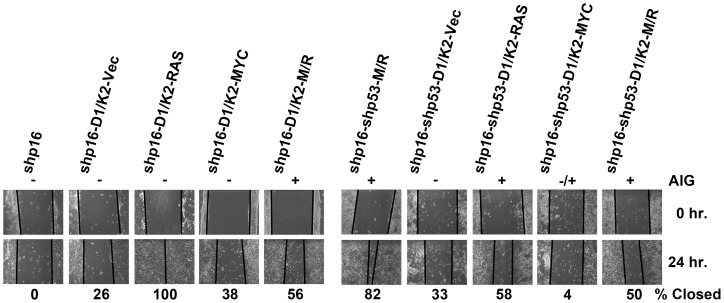
Cell derivatives capable of AIG demonstrate increased motility. Representative pictures of wound healing scratch assay of each HMEC derivative generated to represent all combinations of CCND1/CDK2 (D1/K2) replacements in the transformation protocol. Solid black lines represent cell fronts. Numbers beneath pictures indicate percent closure of scratch at 24 hours. The ability of the cell derivatives to grow anchorage independently is represented above the pictures (+  =  growth, −  =  no growth, −/+  =  intermediate growth).

To determine if transformation to AIG is associated with an aggressive migratory phenotype, each of the cell derivatives was assessed by a wound healing scratch assay. 48R-shp16 and 48R-shp16-shp53-M/R cells were used as negative and positive controls, respectively. Each CCND1/CDK2 replacement combination was tested for the ability to close an artificial wound indicative of a migratory phenotype. Each of the cell derivatives capable of efficient AIG demonstrated 50% or greater closure within 24 hours (**Fig.** **6**). The negative control 48R-shp16 cells and derivatives incapable of AIG (excluding 48R-shp16-D1/K2-RAS) demonstrated less than 33% closure (**Fig.** **6**). Interestingly, cell derivatives expressing oncogenic RAS showed an increased capacity of migration regardless of their ability to grow anchorage independently. These data suggest that cells transformed to AIG demonstrate a greater capacity for motility, but increased migration does not always confer AIG.

## Discussion

**Figure 7:**
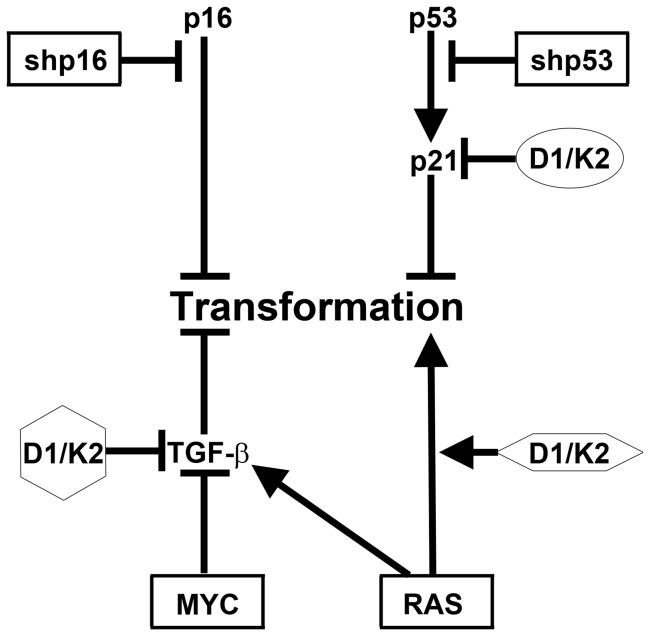
Constitutive CCND1/CDK2 activity replaces p53 inhibition, MYC expression, or oncogenic RAS expression in HMEC transformation. Consistent HMEC transformation to AIG is achieved by stepwise expression of the four molecular perturbations surrounded by rectangles. We have shown that constitutive CCND1/CDK2 (D1/K2) activity can individually substitute for one of three of these events indicated by differing surrounding shapes. Constitutive CCND1/CDK2 activity did not replace any combinations of these events.

Improvements in HMEC culture techniques have facilitated experimental determination of the tumor suppressive and oncogenic pathways that regulate the transformation of normal, finite-lifespan cells. Long-term culture of normal pre-stasis HMEC with functional p16 is now possible [12]. Most finite-lifespan HMEC used currently lack p16 protein expression due to p16 promoter methylation, and harbor many other aberrancies [8], [11], [12]. Employing normal pre-stasis HMEC we have generated a stepwise, genetically-defined model of HMEC transformation to AIG using shp16, shp53, MYC, and RAS expression (**Fig.** **7**), and further expanded upon this model by examining the role of constitutive CCND1/CDK2 activity. The use of a CCND1/CDK2 fusion construct allows the role of CCND1/CDK2 activity to be dissected independently of CCND1/CDK4 activity.

Constitutive CCND1/CDK2 activity could replace, to varying degrees, p53-shRNA, MYC, or RAS in the transformation of HMEC to AIG (**Fig.** **7**). Importantly, the results presented here are consistent with the observation that clinical breast cancers with overexpressed CCND1 typically retain wild-type p53 [25]–[27]. Additionally, the ability of constitutive CCND1/CDK2 activity to replace MYC over-expression is consistent with gene amplification data in clinical breast cancer specimens that show CCND1 and MYC are not typically co-amplified [28]. CCND1/CDK2 complexes have been shown to bind, sequester, and inhibit p21, thereby inactivating the p53-p21 tumor suppressor axis, which may explain how constitutive CCND1/CDK2 activity substitutes for p53 inhibition [22]. Previous reports have shown that MYC phosphorylation induced by CDK2 shifts the cellular response to oncogenic RAS expression from OIS to proliferation and transformation in rat embryonic fibroblasts [29], [30]. We recently identified a p16- and p53-independent RAS-mediated OIS in HMEC that required TGF-β signaling. Constitutive expression of MYC counteracted the TGF-β induced growth arrest, thereby allowing dysregulated RAS to drive HMEC transformation [14]. The observations that CCND1/CDK2 expression can cause TGF-β resistance in mink lung epithelial and MMTVD1-K2 mouse mammary tumor cells, and that CDK2 phosphorylation of MYC abrogates RAS-mediated OIS may explain how constitutive CCND1/CDK2 activity could replace exogenous MYC overexpression and contribute to RAS driven HMEC transformation to AIG in our protocol [22], [23], [29], [30]. Finally, expression of CCND1/CDK2 also partially replaced the ability of RAS to drive transformation. CCND1 is stabilized by RAS effector signaling and implicates CDK activity as one important component of the hyperactive RAS signaling pathways commonly observed in breast cancer [31], [32].

Although constitutive CCND1/CDK2 activity could replace p53 inhibition, MYC expression, or oncogenic RAS expression individually, it could not replace any combinations of these events. As a p53 inhibitor CCND1/CDK2 complexes bind to p21 and may themselves be sequestered, such that these complexes are not available to activate endogenous MYC or substitute for RAS signaling. Therefore, constitutive CCND1/CDK2 activity cannot activate endogenous MYC or replace RAS signaling in the presence of p53. Thus, it does not replace shp53-MYC-RAS, shp53-RAS, or shp53-MYC in the transformation model. CCND1/CDK2 expression replaced RAS less efficiently than the other events likely, because it represents only a fraction of the full signal induced by oncogenic RAS. Therefore, constitutive CCND1/CDK2 activity may not replace both MYC and RAS expression in the transformation model, because it is not powerful enough to replace the full spectrum of oncogenic RAS signaling.

Interestingly, our results demonstrated variable expression levels of the oncogenes in our HMEC derivatives. This variability may be due to the order in which each of the oncogenes was introduced in our protocol, such that the cells may have adapted to the distinct oncogenes differently depending on their order of infection. Indeed, our results showed that replacement of RAS with CCND1/CDK2 generated more AIG when it was introduced after MYC than when it was introduced before MYC. Therefore, the order of genetic defects that occur during tumorigenesis likely affects the evolution of a breast cancer.

Although we demonstrated that constitutive CCND1/CDK2 activity could replace single components in the transformation to AIG, we found that the cells capable of AIG would not form tumors in immunocompromised mice. Upon gross necropsy 12 weeks after injection, we isolated HMEC cell clumps that were vascularized but did not grow substantially. This finding suggests that additional factors may be present in the cells that can suppress tumor formation *in vivo*, or that more severely immunocompromised mice (such as NSG mice) may be necessary for tumor formation with our model.

Luminal breast cancers harboring elevated CCND1 would contain both CCND1/CDK4 and CCND1/CDK2 complexes [21]. Selective inhibitors of CDK4 have been proposed as a therapeutic strategy to suppress constitutive CCND1/CDK4 activity [33]–[36]. Indeed, a recent study demonstrated that luminal-type breast cancer cell lines were the most sensitive to a selective CDK4/6 inhibitor [37]. However, our results studying the unique contributions of constitutive CCND1/CDK2 activity to cellular transformation suggest that targeting CCND1/CDK4 alone does not take into account the consequences of the understudied constitutive CCND1/CDK2 activity we report here. Rather, we propose that a dual treatment strategy targeting CCND1/CDK4 and CCND1/CDK2 complexes will be necessary to effectively treat luminal breast cancers arising from elevated CCND1. A dual treatment strategy would suppress the constitutive, oncogenic CDK activity conferred by dysregulated CCND1expression, and re-engage the cytostatic effects of the p53-p21 and TGF-β pathways. Additionally, we previously demonstrated that activation of wild-type p53 using nutlin-3, an inhibitor of the p53 negative regulator HDM2, could cause target-gene repression that led to growth arrest of CCND1/CDK2 transformed hTERT-immortalized HMEC [24]. Therefore, p53-activating compounds such as nutlin-3 may be effective treatment options for patients with luminal breast cancer due to accumulated CCND1. Combination therapies with specific CCND1/CDK4 and CCND1/CDK2 inhibitors and p53-activators targeted to luminal breast cancer sub-types associated with elevated CCND1 expression should be the most beneficial. Further analysis is warranted and necessary to determine the efficacy of combination therapy in a cohort of patients with luminal breast cancers associated with CCND1 accumulation.

## Materials and Methods

### Cell Lines and culture conditions

Human mammary epithelial cells from specimen 48R were obtained in 1977 from discarded surgical material of a reduction mammoplasty, and provided without identifiers. Use and distribution of the cells is approved under 108H001-1JN13 by the Human Subjects Committee, the Institutional Review Board of Lawrence Berkeley National Laboratories, which holds Office of Human Research Protections Federalwide Assurance number FWA 00006253. Specimen 48R (batch T) and their derivatives were grown in a humidified environment at 37°C with 5% CO_2_ in M87A medium with oxytocin as previously described [12]. Cell growth was determined as population doublings over time. Parental 48R HMEC grew for 19.33 population doublings from passage 6 through passage 14 when the cells encountered the stasis barrier and ceased growth (**Fig.** **2B**). Cell counts were obtained utilizing a Beckman Coulter counter. Population doubling was calculated using the equation, PD = log (cells counted/cells plated)/log2.

### Viral vectors and infection

Passage number for viral infections for each HMEC derivative is represented in **Figure** **S1**. The pBabe-puro-cyclin D1/CDK2 fusion construct (D1/K2) was kindly provided by Brian Law (Department of Pharmacology and Therapuetics, University of Florida, Gainesville, Fl). pBabe-puro (Addgene plasmid 1764) was used as a vector only control. SINhygro-shp16 (shp16) was provided by Dr. Scott Lowe (Cold Spring Harbor Laboratory, Cold Spring Harbor, NY). LNCX2-GFP (VEC), LNCX2-GFP-IRES-RasV12 (RAS), LNCX2-MYC-IRES-GFP (MYC), and LNCX2-MYC-IRES-RasV12 (M/R) were created by subcloning RasV12 from pBabepuro-RasV12 (Addgene plasmid 1768), IRES and GFP from pIRES-GFP (Clontech, 6029-1), and MYC from pwzl-MYC (Addgene plasmid 10674). After successful cloning into LNCX2 (Clontech, 631503) all four constructs were sequence verified. Retroviruses were produced as previously described [38]. Briefly, retroviral vectors were transfected into Phoenix-Ampho cells together with a packaging plasmid encoding the MLV gag, pol, and env genes. The lentiviral vector pLV-shp53-bleo encoding short-hairpin-RNA targeting p53 (shp53) [39] was packaged in 293T cells using the second-generation packaging constructs pCMV-dR8.74 and pMD2G, kind gifts from Didier Trono (University of Geneva, Switzerland). Viral supernatant media containing virus was collected in M87A medium for 24 h, filtered with a 0.22 µm filter, supplemented with 4 μg/mL polybrene, and added to HMEC for infection overnight (18 h). Uninfected cells were removed by selection with G418 (200 µg/mL), puromycin (1 µg/mL), hygromycin (200 µg/mL), or zeocin (200 µg/mL) as appropriate.

### Western blot analysis

Protein was isolated from 48R HMEC at passage 11 and from infected HMEC derivatives at 2 passages after the final infections. Whole cell extracts were prepared by incubating cell pellets in lysis buffer containing 50 mmol/L of Tris (pH 8.0), 150 mmol/L of NaCl, 1.0% NP40, 10 µg/mL of aprotinin, 100 µg/mL of phenylmethane sulfonyl fluoride, 5 µg/mL of leupeptin, 5 µg/mL of pepstatin, and 1 mmol/L of NaVO4. Cell extracts containing equal quantities of proteins, determined by the Bradford method, were separated by SDS-PAGE on precast 4–20% gels (Thermo Scientific, 25204) and transferred to polyvinylidene difluoride membranes (Millipore, IPVH00010). Antibodies to p16 (50.1, SC-9968), p21 (C19, SC-397HRP), p53 (DO-1, SC-126HRP), MYC (9E10, SC-40), and RAS (C-20, CS-520) were obtained from Santa Cruz Biotechnology. Antibody to actin (ACTN05, MS-1295) was from Thermo Scientific. Antibody to Flag (F7425) was from Sigma-Aldrich. Primary antibodies were detected with goat anti-mouse or goat anti-rabbit conjugated to horseradish peroxidase (Rockland Immunochemicals, 610–1302, 611–1302), using enhanced chemiluminescence (Perkin-Elmer, PENEL102001EA) on HyBlot CL film (Denville Scientific, E3018).

### Soft agar assays

Virally infected HMEC derivatives were counted and plated in soft agar 2 passages after the final infections. Cells counted on a Beckman Coulter counter were suspended, 2×10^5^ per 60 mm dish, in 0.6% type VII agarose (Sigma, A4018) and plated onto a bottom layer of 1.2% agar in quadruplicate. Cells were grown for three weeks changing the medium twice weekly until cells were analyzed. Plates were analyzed using Metamorph, in which 5×5 stitched images were counted and multiplied to give colony counts for the entire plate. Graphs were produced in Excel representing the mean of the four counts for each cell line. Error bars represent the calculated standard deviation from the mean of the quadruplicate counts.

### Scratch assays

HMEC derivative cell lines were plated to near confluency in 6-well plates overnight. The next day scratches were made in the cells with a sterile 200 µL pipet tip. Plates were marked on the underside as a landmark for taking pictures. Pictures were immediately taken with a 10× objective on a Nikon eclipse TE2000-S microscope with Metamorph software. Pictures were taken 24 hours later using the marked underside as a landmark for alignment. Cell fronts were approximated with two lines in Photoshop and distances at 0 hour and 24 hours were measured at the midline. The percent closure was calculated as [Distance(0 hr.) – Distance(24 hr.)]/Distance(24 hr.) ×100.

## Supporting Information

Figure S1 **Flow diagram of viral infection strategy and passage number of 48R HMEC and derivatives.** Pre-stasis 48R HMEC were grown from passage 6 until passage 11 (3 passages to stasis) when they were virally infected with the viruses indicated. Arrows represent viral infections of 48R HMEC or its derivatives. The passage number of infection is indicated on the left, and constructs are aligned horizontally with the passage number of infection. Arrows point from the cells infected to the construct(s) that were used for each infection. The derivatives used in each figure are indicated by bars at the bottom. Viral infections include vector control (Vec), shRNA targeting p16 or p53 (shp16) (shp53), CCND1/CDK2 (D1/K2), RAS alone (RAS), MYC alone (MYC), or MYC and RAS together (M/R).
(TIF)
