# Supplementary figures and images for: Constitutive CCND1/CDK2 Activity Substitutes for p53 Loss, or MYC or Oncogenic RAS Expression in the Transformation of Human Mammary Epithelial Cells
Source: PLoS One. 2013 Feb 4;8(2):e53776. doi: 10.1371/journal.pone.0053776 (PMC3563539; doi:10.1371/journal.pone.0053776)

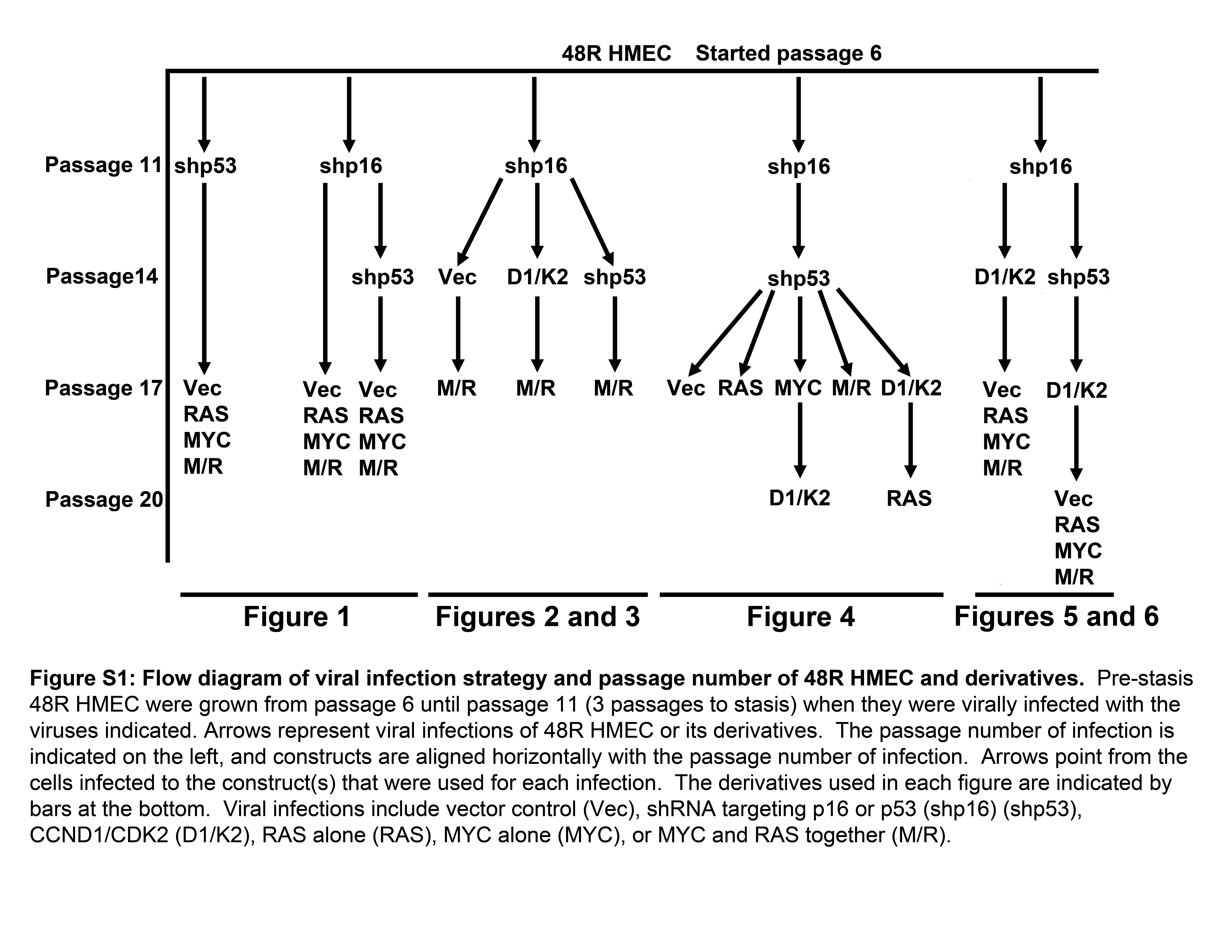

Supplement: Figure S1 — Flow diagram of viral infection strategy and passage number of 48R HMEC and derivatives. Pre-stasis 48R HMEC were grown from passage 6 until passage 11 (3 passages to stasis) when they were virally infected with the viruses indicated. Arrows represent viral infections of 48R HMEC or its derivatives. The passage number of infection is indicated on the left, and constructs are aligned horizontally with the passage number of infection. Arrows point from the cells infected to the construct(s) that were used for each infection. The derivatives used in each figure are indicated by bars at the bottom. Viral infections include vector control (Vec), shRNA targeting p16 or p53 (shp16) (shp53), CCND1/CDK2 (D1/K2), RAS alone (RAS), MYC alone (MYC), or MYC and RAS together (M/R). (TIF) [file pone.0053776.s001.tif]
